# Supplementary material for: P-TRAP: a Panicle Trait Phenotyping tool
Source: BMC Plant Biol. 2013 Aug 29;13:122. doi: 10.1186/1471-2229-13-122 (PMC3848748; doi:10.1186/1471-2229-13-122)
Supplement: Additional file 7 — P-TRAP source code. The Netbeans project that contains the source code of the application. [file 1471-2229-13-122-S7.zip › javadoc.zip!/javadoc/org-bioalg-graphprocessor/org/bioalg/graphprocessor/package-summary.html]

org.bioalg.graphprocessor (GraphProcessor)


JavaScript is disabled on your browser.


- Package
- Class
- Tree
- Deprecated
- Index
- Help

*org.bioalg.graphprocessor 1.0*

- Prev Package
- Next Package

- Frames
- No Frames

- All Classes

# Package org.bioalg.graphprocessor

- Class Summary

  | Class | Description |
  | --- | --- |
  |  |  |
  | --- | --- |
  | BranchModel | A general class for representing branches. |
  | Edge | A general edge class. |
  | EdgeModel | Similar to the `Edge` class but is using vertices instead of points. |
  | Graph | The mathematical graph. |
  | GraphModel | A graph model that can be used to warp xml files, this is more portable than the `Graph` class for both visualizing the results and user manual editing. |
  | Path | A path is the list of vertices starts and ends by two vertices. |
  | Vertex | A graph vertex is a point with a given type. |
- Enum Summary

  | Enum | Description |
  | --- | --- |
  |  |  |
  | --- | --- |
  | Edge.EDGE\_TYPE |  |
  | GraphModel.GraphErrors |  |
  | Vertex.VERTEX\_TYPE | The types of the vertices. |

- Package
- Class
- Tree
- Deprecated
- Index
- Help

*org.bioalg.graphprocessor 1.0*

- Prev Package
- Next Package

- Frames
- No Frames

- All Classes
